# The impact of entrepreneurial activities and college students’ entrepreneurial abilities in higher education—A meta-analytic path

**DOI:** 10.3389/fpsyg.2022.843978

**Published:** 2022-08-02

**Authors:** Jieyu Hua, Kongdi Zheng, Supei Fan

**Affiliations:** School of International Economics and Trade, Nanjing University of Finance and Economics, Nanjing, China

**Keywords:** entrepreneurial activity, higher education, entrepreneurial ability, meta-analysis, moderating variable

## Abstract

The purpose of this study is to quantitatively analyze 34 independent papers collated from both domestic and international literature on the correlation between university entrepreneurial activities and college students’ entrepreneurial abilities by means of meta-regression analysis and to examine in detail the significant factors affecting the entrepreneurial competencies of university students. The study revealed a significant positive relationship between entrepreneurial activities in universities and university students’ entrepreneurial ability, and further explored the extent to which each of the three types of entrepreneurial activities had an impact on college students’ entrepreneurial abilities. Then, the effects of different moderating variables on the relationship are further analyzed. The results show that the type of university, economic development, gender ratio, age structure, and time to publication all significantly moderate the degree of correlation between university entrepreneurial activities and college students’ entrepreneurial abilities.

## Introduction

The concept of “industry–education integration” was first introduced in 1906 by an American professor. In the 1970s and the 1980s, it became widely known in Europe and the United States. Since then, the term “industry-education integration” has been widely used by academics and society and has gradually spread to other parts of the world, such as Asia. This impact was most evident in the United States and Germany. An increasing number of prestigious universities abroad are changing their teaching models and activities to focus more on the practical aspects of teaching and learning and try to involve students more deeply in the classroom. Universities attempt to teach students and share what they have learned and tend to involve them in academic exchange, innovation activities, and entrepreneurship activities. Whether graduates are well equipped for employment and whether they meet the needs of companies is something that universities must pay extra attention to in order to nurture their talent.

Every year, many university students face unemployment immediately after graduation. They receive higher education, have access to better teachers’ resources, own a better platform, and have more opportunities. Students are mostly devoted to working hard to obtain higher grades. However, universities neglect the development of integral skills, such as general literacy, interpersonal communication, and emotional intelligence. Students trained under the original model will only know the knowledge points and theories in the textbooks. When they encounter practical problems that are different from what is taught in books, they lack the ability to deal with practical problems. They will not be able to adapt and solve problems well, and they are more likely to suffer hardships in their work. They are also less innovative, preferring to be set in their ways, and lacking the ability to innovate, often encountering more difficulties, and lacking initiative.

The debate on graduates’ employability and entrepreneurship has been ongoing for decades and has become more intense and conflicting, especially since the 21st century. There is a serious oversupply in the labor market, and universities are struggling to provide graduates that meet the standards required by companies. This is a particularly serious problem.

A study on communities in the United States found that, due to the rise of STEM education, students receiving a higher level of education, especially the training of the basic qualities of entrepreneurship and the ideological enlightenment of entrepreneurship, are conducive to stimulating higher education students’ willingness to start their own businesses. Students who receive better education have an advantage in terms of average wages, and these innovation activities can balance the labor market (Black et al., 2021); Entrepreneurship education in vocational schools can stimulate their potential entrepreneurial enthusiasm, prompt them to seek more avenues, exercise their ability to face failure, not be afraid of setbacks, and play a specific and prominent role in alleviating the unemployment problem of university students (Larbi and Gyedu, 2021).

As a result, a considerable number of universities have made adjustments to their training plans and teaching methods to meet the needs of the times. The integration of industry and education is highly valued by enterprises, universities, and the government. It is becoming a strategy for the development of schools, enterprises, and even the country, with its significant influence and importance. At the same time, the cultivation of innovative talent by enterprises and innovation and entrepreneurship education in universities will now become an obvious trend in the future (Qu, 2021). A study examining the impact of university entrepreneurship education on prospective students’ willingness to engage in entrepreneurship found that university entrepreneurship education tends to stimulate entrepreneurial activities while students are in school or after graduation. Moreover, entrepreneurial activities have a significant effect on students’ self-efficacy (Asimakopoulos et al., 2019). Most students tended to choose a stable and non-adventurous career rather than starting their own businesses. For those who are ready to seek employment, graduates will have more opportunities to seek employment if they have received more adequate and quality education in innovation and entrepreneurship in university (Hassan and Wafa, 2012). They receive more interview offers and tend to be more likely to be hired, with a higher probability of securing a satisfying position with high growth prospects. As such, this paper is not only of research interest but also of policy importance.

Innovation activities in higher education have many forms, such as academic communication, salons, and innovation and entrepreneurship courses, which vary in frequency, quality, breadth, and depth, and the impact of innovation and entrepreneurship education on students can also vary. Students who have received entrepreneurship education tend to find more satisfying jobs at the outset in terms of salary and position and are also promoted more quickly and in a better working environment (Zhang et al., 2014). Universal and compulsory innovation and entrepreneurship education in higher education have a significant positive impact on students’ entrepreneurial intentions and opportunity identification, and students tend to make better choices when faced with important choices (Karimi et al., 2016). Research has found that entrepreneurship education and nurturing have a direct impact on entrepreneurial outcomes and enhance society’s sustainability. However, non-university students are less likely to receive entrepreneurship education and nurturing (Rashid, 2019).

However, scholars have arrived at different conclusions about the relationship between innovation activities in universities and students’ innovation and entrepreneurship. Entrepreneurship in higher education can have a significant impact on students’ willingness to start a business; however, the degree of impact differs significantly between science and arts majors, with science students having a negative impact due to factors such as inadequate emotional intelligence (Maresch et al., 2016).

The experience of receiving innovation and entrepreneurship education has a negative impact on creativity due to the fixed template theory in some entrepreneurship education, which can limit students’ ability to innovate (Tang et al., 2012; Piperopoulos and Dimov, 2015). In addition, there is a clear difference in the role of gender in innovativeness. The study found that men and women have a significantly different propensity to start a business, with men being significantly more willing to do so than women and having a higher success rate in starting a business, according to statistics from the Entrepreneurship Research Society (Pruett, 2012). Therefore, men and women have different roles in the entrepreneurial process; therefore, the adoption of different targeted educational approaches and innovative entrepreneurial activities will become a necessary direction and trend to be studied.

As the current study, different scholars have different conclusions regarding the influence of entrepreneurship activities in higher education on college students’ entrepreneurship, in order to better explore the relationship between the two, this study divides entrepreneurship activities in higher education into three categories: (1) academic communication; (2) science and technology innovation base; and (3) innovation and entrepreneurship courses. College students’ entrepreneurship is divided into four categories: (1) entrepreneurial personality, (2) basic entrepreneurial competencies, (3) core entrepreneurial competencies, and (4) social coping competencies. We used gender, type of university, economic development, time, and age as control variables. An empirical analysis was conducted using a meta-analysis of a sample of 12,873 from 34 papers to scientifically explore the relationship between the two. In the literature review, we explained why we concluded the definition of college students’ entrepreneurial ability, university entrepreneurial activities, and make the hypothesis of the relationship between the two. In addition, we identify the variables that need to be studied in this paper and explain in detail how the study was conducted through the research methods and data source part. In the empirical analysis, we conducted a meta-analysis of the obtained data through CMA3.0. At last, we highlighted the research results and innovation points different from other scholars in this paper in the conclusion part.

## Literature review and research hypothesis

### Entrepreneurial activities affecting college students’ entrepreneurial abilities

Entrepreneurship education is a flourishing field in terms of educational theory and practice (Mao et al., 1992). In 1947, Harvard Business School pioneered the course “Management of Start-up Enterprises” which was the beginning of entrepreneurship education activities in universities worldwide. United Nations Educational, Scientific and Cultural Organization (UNESCO) argues in 1990 that it is the third passport that future generations should have to prove their entrepreneurial and pioneering abilities, which are significant to individuals. It is generally accepted that college entrepreneurship education is a program used to develop entrepreneurial awareness (Wurthmann, 2014). Entrepreneurial activities in higher education include extracurricular competitions, practical socialization, and other activities, in addition to the entrepreneurship education curriculum set by the school (Cui et al., 2021). Tan et al. (2015) defined the content of entrepreneurship activity in higher education as entrepreneurship courses, entrepreneurial lectures, practical entrepreneurship exercises, entrepreneurship competitions, and entrepreneurship training, and then explored the impact of entrepreneurship education on college students’ intention to start a business. In addition, salons held and experts invited to provide on-site guidance are among the education measures commonly taken by schools (Wang and Tang, 2006). Through comprehensive analysis and research, we defined entrepreneurial activities in higher education as academic exchanges related to entrepreneurship, science and technology innovation bases, and innovation and entrepreneurship courses.

Entrepreneurial ability is intellectual capital possessed by entrepreneurs, which is the comprehensive ability of entrepreneurs to successfully start a business or perform their job duties (Thomas et al., 2002). Through a comprehensive analysis, Wang (2019) classified college students’ entrepreneurial ability into innovative thinking, innovation knowledge, entrepreneurial practice ability, and non-intellectual factors. Some scholars also divide students’ entrepreneurial ability according to another perspective, concluding that the ability to associate, the ability to be sensitive to potential profits, and the ability to apply institutional knowledge are the three major elements of entrepreneurial ability (Yang, 2012). This study uses the classification method in the 2014–2015 China College Students’ Employment and Entrepreneurship Development Report as the standard and includes entrepreneurial abilities in four categories and 14 dimensions (Yang, 2015), with the categories being innovative personality, basic entrepreneurial abilities, core entrepreneurial abilities, and social coping abilities. Among them, innovative personality includes courage and boldness, responsibility, practical persistence, confidence, and optimism; basic entrepreneurial abilities include practical ability, learning ability, and analytical ability; core entrepreneurial abilities include innovation ability, opportunity grasping ability, resource integration ability, and leadership ability; social coping abilities include interpersonal skills, teamwork ability, and stress resistance ability.

There is no doubt that higher education has an important and far-reaching impact on students’ careers, and exposing students to entrepreneurship education can encourage them to start their own businesses (Zhang et al., 2014). Previous research by many scholars found that entrepreneurship education activities in universities have a significant impact on college students’ innovation intention (Souitaris et al., 2007; Zhang et al., 2019; Tantawy et al., 2021). Entrepreneurial activities in higher education can not only enhance students’ self-efficacy, increase their knowledge base, and develop their entrepreneurial awareness but also help prepare them for entrepreneurship after graduation. Some scholars have analyzed students’ performance before and after receiving entrepreneurship education training and concluded that such entrepreneurial abilities can be improved by training. Such entrepreneurship education can make a difference to college students’ entrepreneurial behavior and lead to good results in their workplace (Ismail and Zain, 2015). Entrepreneurial activities play a significant role in stimulating students’ interest in entrepreneurship, developing their identification of entrepreneurial opportunities, developing entrepreneurial potential, and strengthening entrepreneurial communication skills (Coduras et al., 2008).

Based on this, this paper proposes the following hypothesis:

*H1*: *There is a significant positive relationship between entrepreneurial activities and entrepreneurial abilities in higher education.*

### Different approaches to entrepreneurial activity affecting entrepreneurial abilities

Innovation and entrepreneurship, a basic course that teaches business thinking and entrepreneurship to school students, includes both how and what to carry out entrepreneurial education (Yang, 2012). Most schools choose to designate entrepreneurship education programs, deploy relevant entrepreneurship mentors, and organize intensive entrepreneurship courses for students (Bergmann et al., 2018). In addition, some colleges try to enhance students’ innovative thinking and practical skills through innovative methods, such as sandbox and virtual simulation teaching methods (Zhang, 2016).

The technology and innovation base, where the university provides an entrepreneurial environment for university students through financial, human, and physical investment, such as research laboratories, is aimed at helping college entrepreneurs complete the transformation of their entrepreneurial projects. The technology and innovation base is an important aspect of entrepreneurial activities in universities to cultivate talent (Yang et al., 2014). Simultaneously, the emergence of makerspaces at Wageningen University and Research Centre (2021) in the Netherlands has provided new ideas for innovative entrepreneurship education systems.

Academic communication related to entrepreneurial activities refers to the extra-curricular activities that the university organizes in addition to compulsory courses, such as academic lectures and inviting experts from outside universities to give lectures and organize entrepreneurial clubs (Wang and Tang, 2006), which can improve students’ innovative and entrepreneurial abilities and help them develop entrepreneurial awareness. Such extracurricular activities are mostly of interest to students, but whether schools take the initiative to provide students with a diverse learning environment for entrepreneurship and create a good entrepreneurial atmosphere is also closely related to students’ initiatives (Yang et al., 2014).

In this study, we investigate the impact of university entrepreneurial activities on students’ entrepreneurial abilities in terms of innovation and entrepreneurship courses, science and technology innovation bases, and academic communication. The hypotheses are as follows:

*H2a*: *There is a significant positive relationship between innovation and entrepreneurship courses and college students’ entrepreneurial abilities.*
*H2b*: *There is a significant positive relationship between the science and technology innovation base and college students’ entrepreneurial abilities.*
*H2c*: *There is a significant positive relationship between academic communication and college students’ entrepreneurial abilities.*

### Potential moderating variables

Entrepreneurial learning is now emerging in several disciplines, both business and science, and entrepreneurship education is a fundamental part of college activities, which varies from one college to another (Ceresia, 2018). Comprehensive colleges combine arts and science disciplines and facilitate interdisciplinary collaboration, with the learning outcomes can be assessed in five categories: “strongly integrated,” “sub-integrated,” “weakly practical,” and “weakly emotional” (Wu, 2016). In the professional category, universities with obvious professional characteristics and close connections between majors and social and economic development and entrepreneurship education have been gradually highlighted (Zhao, 2014). Students in science disciplines tend to be more willing to face risks and take consequences than those in other disciplines (Krueger and Dickson, 1994), which is categorized in entrepreneurial abilities as courageous daring in an innovative personality. Roger and Martyn (1999) found significant differences in entrepreneurial intentions between students with different degrees, while Hassan and Wafa (2012) argued that students who study in science-based disciplines gain higher levels of increased innovation and have higher entrepreneurial intention than students in other disciplines.

This study investigates the impact of entrepreneurial activities in higher education on students’ entrepreneurial abilities at both general and professional universities. The hypotheses are as follows:

*H3*: *The relationship between entrepreneurial activities and college students’ entrepreneurial abilities is more significant in professional colleges than in comprehensive ones.*

National and international studies have shown that gender is an important factor that cannot be ignored when researching and assessing entrepreneurial competencies (Bergmann et al., 2018). In terms of the gender factor, the link between gender and entrepreneurship has been more intensively researched in developed countries (De Vita et al., 2014). Research shows that gender differences can have an impact on entrepreneurs in contemporary society, including the fact that men have a wider range of entrepreneurial options and that women will have more family responsibilities than men (Yang et al., 2017). However, the entrepreneurial abilities of men and women do not differ as much as they do when they are of the same age and educational background, and in the context of receiving entrepreneurial activities at university. Zhu et al. (2021) suggest a moderating effect of gender on the association between entrepreneurial values and entrepreneurial self-efficacy and between entrepreneurial self-efficacy and entrepreneurial intention. Other studies have shown that by implementing the OzGirls entrepreneurship program, women’s entrepreneurial intentions increased significantly, and they showed a high level of competence and entrepreneurial potential in entrepreneurship education activities (Shahin et al., 2021). Vukmirović (2019) argues that offering learning methods about female entrepreneurship or organizing lectures by successful female entrepreneurs can significantly influence young women’s entrepreneurial intentions and behavior. Huang et al. (2017) find that women are more inclined to go into business than men, and explain this phenomenon from the perspective that modern women are trying to break traditional social perceptions.

Therefore, based on the above analysis, we explore the impact of entrepreneurial activities in higher education on college students’ entrepreneurial abilities from a gender perspective and propose the following hypotheses:

*H4*: *The relationship between entrepreneurial activities in higher education and college students’ entrepreneurial abilities is more significant for females than for males.*

The entrepreneurial environment plays a non-negligible role in the development of entrepreneurial abilities among college students, and the economic development status is an important factor in the entrepreneurial environment (Fang et al., 2021). Domestic and international studies have assessed the entrepreneurial activities of entrepreneurs from different economies and found that such abilities and entrepreneurial intentions differ significantly between economically developed and developing countries (Munir et al., 2019). For developed countries, where technology and talent systems are well established, research has found that high-GDP countries show a disincentive to engage in early and mature entrepreneurial activity (Zhao et al., 2012). Students are exposed to and learn about the entrepreneurship process from a young age through a variety of channels. However, the development of entrepreneurship education activities in college does not lead to significant improvements in students’ entrepreneurial skills, and entrepreneurial motivation is somewhat lacking. In developing countries, students are more willing to start their own businesses because of their highly economically upside and supportive national policies. If universities promote entrepreneurial activities and provide adequate support, students’ entrepreneurial abilities are greatly enhanced (Salamzadeh et al., 2013). Further, research on Nigeria shows that entrepreneurship education in higher education is gradually flourishing and becoming a beneficial tool for social and economic development, with far-reaching strategic implications for developing countries (Sanusi et al., 2017).

Therefore, based on the above analysis, we explore the impact of entrepreneurial activities in higher education on college students’ entrepreneurial abilities in terms of their level of economic development, and propose the following hypothesis:

*H5*: *The relationship between entrepreneurial activities in higher education and college students’ entrepreneurial abilities is more significant in developing countries than in developed countries.*

UNESCO’s article (2016) *Rethinking education: A shift towards the idea of ‘global common good?’* is another major education report published after 1996, and its content has led to new global thinking about how and what education is taught in schools. The report states that the world is changing, and that education needs to change even more. Schools should focus on developing students’ comprehensive skills, fostering critical thinking, and on changing the current educational model. Since 2016, entrepreneurship education has also gained more attention, and the content of entrepreneurship education tends to be diverse.

Therefore, we chose year 2016 as the time point to explore the impact of entrepreneurial activities on college students’ entrepreneurial abilities with the following hypothesis:

*H6*: *The relationship between entrepreneurial activities and college students’ entrepreneurial abilities is more significant in the literature published after 2016 compared to before 2016.*

In general, students typically enter college campuses at the age of 18 and complete their undergraduate credits after the age of 22–23, at which point they are faced with various choices of entrepreneurship, employment, or further education. As students grow older and continue to learn and engage with society, their accumulation of experience, psychological state, and perceptions of society and themselves change significantly during this process, which in turn changes their entrepreneurial abilities and behaviors (Marvel et al., 2016). In addition, some studies have shown that entrepreneurial interest among junior undergraduates is greater than at the master’s or doctoral level, which can be explained by the fact that students’ willingness to start a business and their ability to absorb knowledge is higher (Hahn et al., 2017), while postgraduate students have a more traditional approach to entrepreneurship education (McKeown et al., 2006).

Therefore, based on the above analysis, we explore the impact of entrepreneurial activities in higher education on college students’ entrepreneurial activities from an age perspective and propose the following hypotheses:

*H7*: *The relationship between entrepreneurial activities and college students’ entrepreneurial abilities is more significant before age 23 than after age 23.*

**FIGURE 1:**
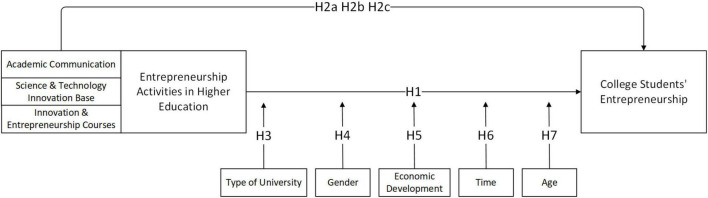
The overall study hypothesis framework.

Based on the above literature review and research hypothesis, the overall study hypothesis framework for this study was obtained, as shown in Figure 1.

## Research methodology and data sources

### Research methodology

In the current study, different scholars have obtained different research results regarding the influence of innovation activities on the cultivation of innovative and entrepreneurial talent in universities. To better explore the relationship between the two, this study adopted a meta-analysis method. This is because the method of meta-analysis is not only a qualitative description and summary analysis of the literature but also a quantitative research analysis of the collected and screened literature, which can be re-tabulated and re-analyzed, and can reduce the selectivity bias and model missetting bias of the original literature (Bakucs et al., 2013; Doanh and Bernat, 2019). The selection of several papers with different findings corresponds to a larger sample size, which can, to some extent, improve the original problem of a small sample size. By collecting the literature, we found that the current study provides a certain empirical basis for meta-analysis. A certain database exists. We will use meta-analysis to further explore the impact of university innovation activities on innovation and entrepreneurship talents and strive to draw more accurate and valid conclusions.

### Literature search and selection

The first search was based on keywords such as integration of industry and education, innovation activities, university research activities, and the cultivation of innovative and entrepreneurial talent. In order to make the search more comprehensive and professional, covering both domestic and international articles, and to overcome the limited inclusion of texts in a single database, we conducted searches in Chinese and English databases such as China Knowledge Network, Wanfang Database, Elsevier, Springer Link, Taylor&Francis, MDPI, and Emeraldinsight. The literature was published between 2008 and 2021. The specific search and screening process was as follows: (1) 658 articles were searched in the database based on the above keywords; (2) 342 articles with duplicate titles and duplicate studies were removed; (3) 173 articles that did not include the correlation coefficient tables were removed; (4) 143 articles that did not meet the inclusion criteria were removed; (5) 15 articles that met the requirements after review were added; and (6) 24 articles with incomplete data were excluded. After the above six steps, 32 articles were identified for the meta-analysis.

### Literature coding and data processing

**FIGURE 2:**
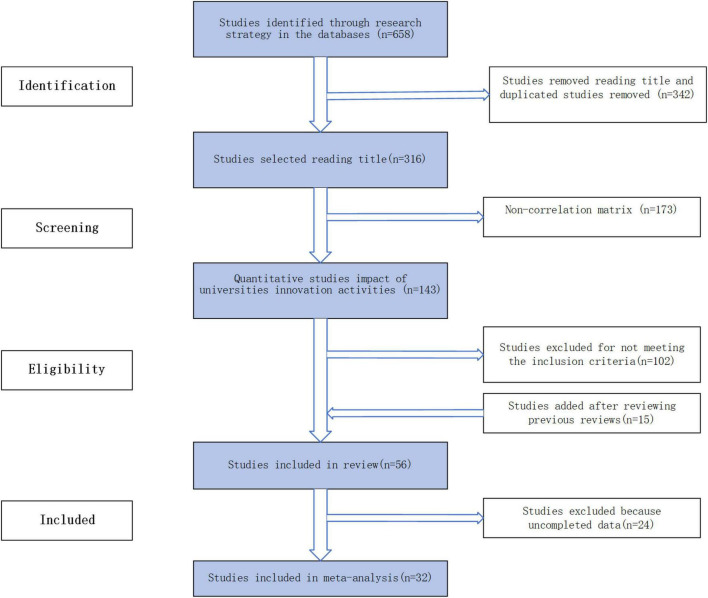
Stages in the process for selecting studies for the meta-analysis.

This study used CMA3.0 software to conduct the meta-analysis, and the required literature data were the sample size and correlation coefficient r. After several searches and screenings, 32 empirical articles containing correlation coefficient tables were identified, with a total sample size of 12,873, an average sample size of 402, a maximum sample size of 1428, and a minimum sample size of 109. The final literature data were imported into the CMA3.0 software, and after several analysis operations, the final results were obtained. Thirty-two empirical articles can be found in Table 1 and detailed results are shown in Figure 2.

**TABLE 1:** Summary of basic information of the included literature.

First author, published year	Correlation coefficient	Sample size	Type of university	Economic development	Entrepreneurial activities	Categories of entrepreneurship
Cui et al., 2021	0.551	1428	Comprehensive	Developing	Academic communication	Core entrepreneurial competencies
Tantawy et al., 2021	0.35	109	Comprehensive	Developed	Science and technology innovation base	Entrepreneurial personality
Ahmed et al., 2020	0.27	348	Comprehensive	Developing	Innovation and entrepreneurship courses	Basic entrepreneurial competencies
Maheshwari and Kha, 2022	0.246	401	Comprehensive	Developing	Innovation and entrepreneurship courses	Entrepreneurial personality
Zhang et al., 2019	0.44	411	Comprehensive	Developing	Academic communication	Entrepreneurial personality
Zhang et al., 2014	0.25	494	Comprehensive	Developing	Science and technology innovation base	Entrepreneurial personality
Hahn et al., 2017	–0.0012	427	Comprehensive	Developed	Science and technology innovation base	Core entrepreneurial competencies
Maheshwair, 2021	0.385	164	Comprehensive	Developing	Innovation and entrepreneurship courses	Entrepreneurial personality
Rosiqie-Blaso et al., 2017	0.329	1126	Professional	Developing	Innovation and entrepreneurship courses	Core entrepreneurial competencies
Lavelle, 2018	0.189	321	Comprehensive	Developing	Innovation and entrepreneurship courses	Core entrepreneurial competencies
Puni et al., 2018	0.39	357	Comprehensive	Developing	Innovation and entrepreneurship courses	Core entrepreneurial competencies
Zhao and Wang, 2016	0.408	350	Comprehensive	Developing	Innovation and entrepreneurship courses	Core entrepreneurial competencies
Lagula et al., 2019	0.37	559	Comprehensive	Developed	Academic communication	Basic entrepreneurial competencies
Herman, 2019	0.179	138	Professional	Developing	Innovation and entrepreneurship courses	Entrepreneurial personality
Westhead and Solesvik, 2015	0.23	189	Professional	Developed	Innovation and entrepreneurship courses	Core entrepreneurial competencies
Hassan et al., 2020	0.475	334	Professional	Developing	Innovation and entrepreneurship courses	Core entrepreneurial competencies
Piperopoulos and Dimov, 2015	–0.01	114	Professional	Developed	Science and technology innovation base	Core entrepreneurial competencies
Tang et al., 2012	–0.19	400	Comprehensive	Developed	Science and technology innovation base	Core entrepreneurial competencies
Saeed et al., 2015	0.3	805	Comprehensive	Developing	Innovation and entrepreneurship courses	Entrepreneurial personality
Mahmoud et al., 2020	0.332	260	Comprehensive	Developed	Innovation and entrepreneurship courses	Core entrepreneurial competencies
Yordanova et al., 2020	0.16	337	Comprehensive	Developing	Science and technology innovation base	Social coping competencies
Martin-Rios et al., 2019	0.22	273	Comprehensive	Developing	Science and technology innovation base	Core entrepreneurial competencies
Madrid-Guijarro et al., 2021	0.524	267	Professional	Developing	Science and technology innovation base	Social coping competencies
Lee et al., 2020	0.544	169	Professional	Developed	Academic communication	Core entrepreneurial competencies
Zhang and Groen, 2021	0.221	1202	Professional	Developed	Academic communication	Basic entrepreneurial competencies
Li and Wu, 2019	0.557	221	Comprehensive	Developing	Innovation and entrepreneurship courses	Core entrepreneurial competencies
Asimakopoulos et al., 2019	0.284	208	Professional	Developed	Innovation and entrepreneurship courses	Core entrepreneurial competencies
Oyugi, 2011	0.464	255	Comprehensive	Developing	Innovation and entrepreneurship courses	Core entrepreneurial competencies
Turker and Senem Sonmez, 2009	0.226	300	Comprehensive	Developing	Innovation and entrepreneurship courses	Core entrepreneurial competencies
Paray and Kumar, 2020	0.329	309	Comprehensive	Developing	Innovation and entrepreneurship courses	Core entrepreneurial competencies
Bellucci and Pennacchio, 2016	–0.28	452	Comprehensive	Developed	Science and technology innovation base	Basic entrepreneurial competencies
Fan-Chuan et al., 2020	0.431	145	Comprehensive	Developing	Science and technology innovation base	Core entrepreneurial competencies

## Analysis of empirical

### Overall effect

(1) Publication bias

**FIGURE 3:**
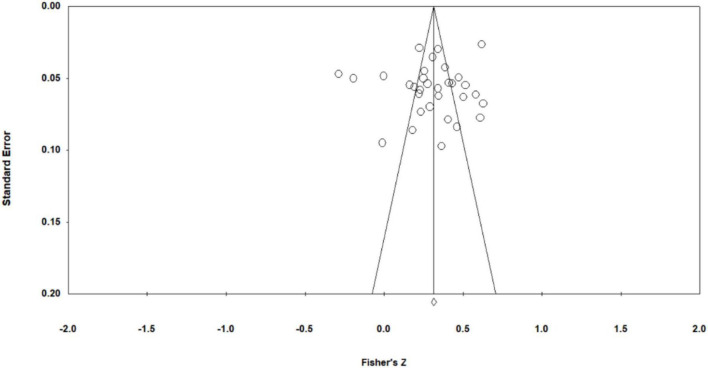
Funnel Plot of Standard Error by Fisher’s Z.

Before conducting the meta-analysis, we conducted a bias test of the effect values to ensure the rigor of the article’s results. The funnel plot as shown in Figure 3 was generated by CMA3.0 software. According to the figure, it can be found that most of the data used in this paper are clustered at the top of the chart, diverged downward along both sides of the curve and distributed evenly around the average effect value, with only a few data deviating from the funnel plot. According to the principle of the funnel plot, if the points are distributed at the bottom of the funnel plot and show the form of dispersion around, the sample size is small, the precision is low, and the degree of bias is high. If the points are gathered at the top of the funnel plot and clustered toward the middle, it means that the sample size is larger, the precision is higher, and the degree of bias is low. In view of this, the risk of publication bias in this study was small.

(2) Heterogeneity tests

The heterogeneity test, also known as the chi-square test, aims to check whether the results of each independent study are mergeable, and whether the mergeability and parallelism among multiple independent samples is a prerequisite for comprehensive analysis. Therefore, the heterogeneity test is an essential part of the Mate analysis. Generally speaking, there are two methods for testing heterogeneity: the Q-value test compares the Q-value with the number of effect values S (the number of integrated studies included); if Q ≤ S-1, both the random and fixed models can be used, and if Q > S-1, only the random effect model is used; the second is the I^2^ test, which reflects the proportion of heterogeneity in the total variance of effect sizes, and the larger the I^2^ value, the greater the heterogeneity. That is, when I^2^ ≥ 50%, it indicates the existence of heterogeneity, and a random effect model should be used; when I^2^ < 50%, it indicates that heterogeneity is not strong and a fixed-effect model should be used. This study combines the above two methods, and the test results are presented in Table 2. Finally, the Q value was 590.425 (*p* ≤ 0.001), which is far more than the comparison value32; I^2^ was 94.75%, which is also far more than 50%, both of which indicate that there is large heterogeneity in the sample, and a random effect model should be used. Meanwhile, it can be seen from Table 2 that the random combined effect value of the literature is 0.296(*p* ≤ 0.001); that is, the overall correlation coefficient between entrepreneurial activities and college students’ entrepreneurial abilities is 0.296. Combined with the I^2^ value, it is clear that the degree of variation due to random error is only 6.25%, and H1 is verified.

**TABLE 2:** Heterogeneity test.

Model	Combined effect value	Number studies	95%CI		Z-value	Heterogeneity			
			Upper limit	Lower limit		df	I^2^	Q-value	*P*-value
Fixed	0.305		0.321	0.289	35.623				
		32				31	94.75	590.425	0.000
Random	0.296		0.364	0.224	7.731

(3) Hypothesis testing of overall effects

We studied the influence of entrepreneurial activities on students’ entrepreneurial abilities from three dimensions: innovation and entrepreneurship courses, science and technology innovation base, and academic communication. The results are shown in Table 3. It is hereby declared that some literatures do not strictly and explicitly state the classification according to entrepreneurship courses, base construction, and academic communication, but they can be incorporated into a certain dimension according to relevant content. Therefore, we divide them into corresponding dimensions according to the specific content of the articles. As shown in Table 3, the correlation coefficients of innovation and entrepreneurship courses, science and technology innovation base, academic communication, and students’ entrepreneurial abilities were 0.334 (*p* ≤ 0.001), 0.152 (*p* = 0.091), and 0.430 (*p* ≤ 0.001), respectively. Thus, all have a positive correlation, and the hypotheses of H2a, H2b, and H2c were verified. This result also indicates that most scholars agree that the development of entrepreneurial activities and their related forms in colleges has a certain promotional effect on the improvement of students’ entrepreneurial abilities. Therefore, considering the current social demand or long-term social development, colleges should actively carry out related innovative and entrepreneurial activities to improve students’ entrepreneurial abilities and cultivate high-quality talent.

**TABLE 3:** Global effect hypothesis testing.

Group	Combined effect value	Number studies	Sample size total	Q-value	df	*P*-value	95% CI	
							Upper limit	Lower limit
IEA	0.328	32	12873	590.425	31	0.000	0.370	0.285
IECE	0.334	17	6086	62.429	16	0.000	0.379	0.287
STIB	0.152	10	3018	211.599	9	0.091	0.318	–0.024
AC	0.430	5	3769	108.310	4	0.000	0.563	0.275

IEA, innovation and entrepreneurship activities in universities; IECE, innovation and entrepreneurship course education; STIB, science and technology innovation base; AC, academic communication.

### Moderating effects—Meta regression analysis

The type of university, age, gender, year2016, and the economic development were used as moderating variables. To make the relevant results clearer, we adopted 0–1 coding for the literature as a way to illustrate the effects of each potential moderating variable on the relationship between entrepreneurial activity in higher education and college students’ entrepreneurial abilities, and the results are shown in Table 4.

**TABLE 4:** Adjust the effect.

Varible	Number studies	Combined effect value	95% CI		Z-value	Heterogeneity			
			Upper limit	Lower limit		df	I^2^	Q-value	*P*-value
H3: School type								590.003	≤0.001
Professional	9	0.324	0.416	0.226	6.202	8	88.951	72.407	≤0.001
Comprehensive	23	0.286	0.376	0.190	5.675	22	95.75	517.596	≤0.001
H4: Gender								551.308	≤0.001
Female	9	0.365	0.474	0.244	5.621	8	91.974	99.67	≤0.001
Male	16	0.280	0.378	0.176	5.113	15	94.575	276.509	≤0.001
H5: Country								429.285	≤0.001
Developing	21	0.355	0.412	0.296	10.898	20	89.510	190.663	≤0.001
Developed	11	0.174	0.321	0.019	2.198	10	95.809	238.623	0.028
H6:Year 2016								588.090	≤0.001
Before	10	0.208	0.392	0.007	2.030	9	97.895	427.483	0.042
After	20	0.330	0.0392	0.266	9.454	19	88.127	160.027	≤0.001
H7:Age 23								509.975	≤0.001
More than 23	2	0.239	0.370	0.099	3.296	13	95.908	317.702	≤0.001
Less than23	14	0.337	0.413	0.256	7.708	15	92.145	190.950	≤0.001

According to the analysis of the results in Table 4, firstly, the correlation coefficient between entrepreneurial activities and students’ entrepreneurial abilities in professional universities is 0.324 (*p* ≤ 0.001), which is larger than the correlation coefficient between them in comprehensive universities 0.286 (*p* ≤ 0.001), and the heterogeneity is significant (Q = 590.003, *p* ≤ 0.001), indicating that in the influence of entrepreneurial activities on students’ entrepreneurial abilities, the correlation between entrepreneurial activities of professional universities and students’ entrepreneurial abilities is stronger than that of comprehensive universities, and the research hypothesis H3 is supported by the test. Second, the correlation coefficient of 0.365 (*p* ≤ 0.001) when the number of female participants exceeds 50% is greater than the correlation coefficient of 0.280 (*p* ≤ 0.001) when the number of female participants is less than 50%, and the heterogeneity is significant (Q = 551.308, *p* ≤ 0.001), indicating that the correlation between entrepreneurial activities and entrepreneurial abilities is stronger for women compared to men, and hypothesis H4 is supported by the test. Third, the effect value of 0.355 (*p* ≤ 0.001) between entrepreneurial activities and students’ entrepreneurial abilities in developing countries is higher than the effect value of 0.174 (*p* ≤ 0.05) between the two in developed countries, and the heterogeneity is significant (Q = 429.285, *p* ≤ 0.001), indicating that the correlation between entrepreneurial activities and students’ entrepreneurial abilities in developing countries is the research hypothesis H5 was supported by the test. Fourth, the effect value of 0.208 (*p* ≤ 0.05) for both before 2016, when the educational perspective of “global common good” was not formed, is smaller than the effect value of 0.330 (*p* ≤ 0.001) after 2016, and the heterogeneity is significant (Q = 588.090, *p* ≤ 0.001), indicating that the relationship was more significant in the literature published after 2016 compared to before 2016, and hypothesis H6 was supported by the test. Fifth, the correlation coefficient between the two was 0.337 (*p* ≤ 0.001) for activity participants younger than 23 years old and 0.239 (*p* ≤ 0.001) for participants older than or equal to 23 years old, and the heterogeneity was significant (Q = 509.975, *p* ≤ 0.001), indicating that entrepreneurial activities in higher education before 23 years old were more significantly related to students’ entrepreneurial abilities. This relationship was more significant, and Hypothesis H7 was supported by the test.

A summary of the results of the theoretical hypothesis testing based on the above results is shown in Table 5.

**TABLE 5:** Total summary results of the theoretical hypothesis.

Assuming number	The inspection results
H1: There is a positive correlation between entrepreneurial activities and entrepreneurial abilities in higher education	Support
H2a: There is a positive correlation between innovation and entrepreneurship education and college students’ entrepreneurship abilities.	Support
H2b: There is a positive correlation between science & technology innovation base and college students’ entrepreneurial abilities.	Support
H2c: There is a significant positive correlation between academic communication and college students’ entrepreneurial abilities.	Support
H3: The relationship between entrepreneurial activities and college students’ entrepreneurial abilities is more significant in professional colleges.	Support
H4: The relationship between entrepreneurial activities and college students’ entrepreneurial abilities is more significant for females.	Support
H5: The relationship between entrepreneurial activities and college students’ entrepreneurial abilities is more significant in developing countries.	Support
H6: The relationship between entrepreneurial activities and college students’ entrepreneurial. abilities is more significant in the literature published after 2016.	Support
H7: The relationship between entrepreneurial activities and college students’ entrepreneurial abilities is more significant before the age of 23.	Support

## Conclusion

Despite the importance of entrepreneurship education, there is surprisingly little research on it (Zhang et al., 2014). In particular, the influence of entrepreneurial activities on students’ entrepreneurial ability has been ignored in the existing research. This study presents a comprehensive analysis of past studies on the impact of entrepreneurial activities on college students’ entrepreneurial abilities through 32 valid papers, adopts the component analysis method, introducing university type, gender, age structure, and other regulatory variables, comprehensive research on the impact of entrepreneurship education in colleges and universities on the college students’ entrepreneurial ability, and in-depth discussion on the relationship between the two, the following conclusions are drawn.

### Overall analysis

According to Lipsey and Wilson’s study, an effect value with a correlation coefficient greater than or equal to 0.25 is considered to be moderately correlated, while an effect value greater than or equal to 0.40 is considered to be highly correlated. The influence value of entrepreneurship activities and entrepreneurial ability of college students is 0.328, which is significantly positive correlation. It can be seen that college students’ Innovation and entrepreneurship activities are an important way to improve their entrepreneurial ability. Generally speaking, entrepreneurship includes two basic and important factors, namely entrepreneurial spirit and entrepreneurial skills. The so-called entrepreneurial spirit refers to the adventurous spirit, enterprising spirit and innovative spirit reflected in the practice of career development. It includes independent spirit and courage. Independent spirit is one of the most important qualities that an entrepreneur has. An entrepreneur never feels accomplished in a company with new ideas are not appreciated, where he/she can’t apply those ideas, an entrepreneur wants to be its own boss, to take risks and higher responsibilities. Entrepreneur must also have courage to implement the ideas, to follow them, to believe, to realize the difference. Sometime, all of us have brilliant ideas, but very few of us have the courage to assume and follow them, and for the other ones those ideas never become true (Zamcu, 2013). And entrepreneurial ability refers to the comprehensive ability of organize resources and create something new (Schumpeter, 1934). In Stevenson’s vision “an entrepreneur has the ability to identify and develop business opportunities.” At the same time, entrepreneurship is a very practical and complex activity, which requires a lot of social resources. By participating in Innovation and entrepreneurship activities in universities, college students can effectively enhance their entrepreneurial inspiration, obtain entrepreneurial resources, and improve their entrepreneurial attitude (Souitaris et al., 2007). In this way, students learn in practice and return in practice, so as to enhance the spirit of entrepreneurship and entrepreneurial skills. However, combined with Carrre and Sequeira’s findings, students’ previous entrepreneurial experiences could be positive or negative. When the legal system is not sound, entrepreneurial activities are risky and the return is not guaranteed, and the failed entrepreneurial experience may have negative or even greater impact. When students see the negative consequences of their friends’ entrepreneurial failure (bankruptcy, life pressure, and suicide), they may develop stronger resistance to entrepreneurship, and this effect seems to be stronger than the effect of entrepreneurship education. Although the results of entrepreneurial contact are surprising, no attention has been paid to it, and no article has elaborated the relationship between the two. Therefore, it is particularly important to study the issue of entrepreneurial contact in depth. Therefore, in the early stage of deciding to carry out Innovation and entrepreneurship activities, colleges and universities must seriously consider the environment of activities, timely strengthen the construction of teaching staff, and provide students with professional and accurate guidance as far as possible.

There is also a significant positive correlation between the three dimensions of higher education entrepreneurial activities and entrepreneurship. Entrepreneurship education is to promote entrepreneurship by influencing attitudes, values and the general community culture. This aim is the driving force behind all other objectives, namely start-ups, self-employment, jobs creation, knowledge advancement and skill development (Mwasalwiba, 2010). In most countries, the long-term general education to the national personality development, lead to students lack of innovative entrepreneurial awareness, innovation, entrepreneurship education will surely help to those who will or are studying entrepreneurship of college students according to their own conditions to the various needs of the knowledge and ability, promote the school to complete the task of cultivating talents. The focus of the science and technology innovation base is to train students’ innovative thinking, encourage them to go out of class, combine theory with practice, improve students’ self-thinking ability and teamwork ability through activities, and improve their comprehensive ability. At the same time, quite a number of innovative entrepreneurial base will provide financial support, due to the lack of social resources, college students even with a good product idea, but financial investment institutions will not invest in the early stage of industrialization of scientific and technological achievements, single financing approach makes it difficult to commercialize products.The establishment of science and technology innovation base has solved part of the financial problem and made many product ideas realize commercialization. It is obvious that effective entrepreneurial base can improve students’ entrepreneurial ability. Effective academic exchange can not only enable individuals to acquire certain entrepreneurial knowledge, but also create a good entrepreneurial atmosphere. The guidance of experienced experts or teachers can greatly reduce the mental burden of entrepreneurs. More importantly, it can timely help entrepreneurs to provide consulting and guidance on the operation and management of the company, which greatly improves their entrepreneurial ability and the viability and development potential of start-ups.

### Moderating effects

First of all, the impact of human capital factors such as education level, political status and social network on students’ cognition of employment varies with the types of schools they attend. College students’ cognitive level on their employment ability affects whether they can correctly make self-evaluation and whether they can meet their own career development, and targets under the market condition (Li and Fan, 2018). College students’ cognition of employment will directly affect their actual employment activities. This paper finds that compared with comprehensive colleges, entrepreneurial activities in higher vocational colleges are more likely to improve the entrepreneurial ability and entrepreneurial willingness of college students, indicating that entrepreneurial activities in higher vocational colleges are more rewarding. This finding has not been further studied in many similar articles. Specialized colleges and universities have the characteristics of obvious specialization and concentration of research direction. Teachers in schools are highly interactive, and participating teachers can provide students with more accurate and professional guidance. These universities have also been training and transporting employment talents for relevant industries, and have a certain industry reputation. Such an environment not only allows students to have a specific industry network, but also facilitates whether they want to start a business in that industry. Based on this, the government should guide and support students with high entrepreneurial enthusiasm and strong action ability in professional colleges and universities. At the same time, stimulate the entrepreneurial enthusiasm of comprehensive colleges students. Although the research found that such students have low entrepreneurial intention and action motivation, they still have high potential and strong ability, which should be paid attention to. Meanwhile, the degree of social network has subtle influence on entrepreneurial ability and entrepreneurial intention. A good social network is conducive to creating a good entrepreneurial atmosphere, so it is crucial to establish certain social networks on campus, such as school-enterprise cooperation.

Second, the relationship between entrepreneurial activity and entrepreneurial ability is more significant for women than for men. This result is contrary to the research conclusion of Yuexiu (2013). The findings underscore the need for the current public debate on the need for more female role models (Zhang et al., 2014). It is important for governments and educators to recognize that entrepreneurial activity is often associated with masculinity, and that the lack of female role models seems to reinforce these stereotypes. Therefore, it is necessary for us to take steps to break traditional stereotypes and cultivate entrepreneurship in a way that is more attractive to women. However, in the course of our research, we found a correlation between gender and years of education, which may led to dexiations in the above conclusions. When the number of years of education is shorter, men are more willing to start a business than women. With the improvement of education level, the age of starting a business reaches about 17 years, which means that men and women have almost the same desire to start a business.

Third, entrepreneurial activities in developing countries have a more pronounced effect on students’ entrepreneurial abilities than those in developed countries. Factors such as poor infrastructure and scarce educational resources are both disadvantages, but they also stimulate the desire for entrepreneurial skills and corresponding entrepreneurial opportunities for developing countries as well as students. In the context of the globalization of education, the support of relevant policies introduced by the government also provides guarantees for more institutions to carry out entrepreneurial activities and enhance the effectiveness of their implementation. In addition, developed countries have accumulated considerable theoretical and practical experience in the implementation of entrepreneurship activities in colleges and universities, and developing countries can achieve similar or even better results with less learning costs. Therefore, for developing regions, it is now the best time to vigorously organize entrepreneurship activities. However, it is worth warning that directly borrowing advanced experience and technology from developed countries can certainly bring short-term profits, but will definitely contradict self-generated development in the long run. The country should clarify its strengths and weaknesses, and identify the most suitable path for itself.

Fourthly, the relationship between entrepreneurial activities of colleges and universities and entrepreneurial ability of college students is more significant than before in the literature published after 2016. This may be because in 2016, UNESCO reflected on the nature of education and published the book rethinking Education: Transforming Perceptions of the Global Common Good, which has had a broad impact on global education. The introduction to the report states that education should be based on humanitarianism, with respect for life and human dignity, equal rights, social justice, cultural diversity, international solidarity and shared responsibility for a sustainable future. This establishes the idea that education is a global good, taking a more mature and flexible approach to lifelong learning in a holistic way, providing all the opportunities to realize their potential and achieve a sustainable future. This will provide a good foundation for future exchanges of entrepreneurial activities between different regions and create a good social atmosphere. However, due to the reform of education policies, the existing literature on the impact of entrepreneurial activities on entrepreneurial ability is basically positive, while the literature on the negative correlation is weak. In this case, it is difficult to publish papers with negative correlation, which challenges the authority of the conclusion.which is consistent with the research results of Huang et al. (2017), but has not been shown in other relevant literatures. However, colleges and universities still need to take advantage of the current policy advantage to encourage entrepreneurship and innovation, increase investment in education, support college students to start their own businesses, and solve the employment problem of some college students.

Fifth, the correlation between entrepreneurial activities in higher education and entrepreneurial ability of college students is more significant before the age of 23 than after it. In fact, it is not hard to understand that students’ learning ability, willingness to learn and self-efficacy peak before the age of 23. At the same time, most students are in the fourth year or graduation stage of their undergraduate studies, which is the stage of contact with society. In addition, social work has been in a state of saturation, most school governments have been encouraging students to start their own business policy attitude, which has a certain impact on the entrepreneurial ability of students. However, during this period, the enterprises founded by students are still in the embryonic stage. They lack experience and financial connections, and are easily swallowed up by large companies. How to support the development of these enterprises correctly and effectively becomes an important problem.

## Limitation of research

In this study, we strictly followed the criteria of meta-analysis to screen the literature, conduct data analysis and integration to explore the relationship between entrepreneurial activities and the influence of college students’ entrepreneurial abilities, and introduce the corresponding moderating variables, but there are still shortcomings.

First, meta-analysis itself has certain limitations. Only when there is no effect modification can there be a specific data interpretation of the combined effect size, but paradoxically, it is impossible to determine whether there is effect modification. In other words, the resulting combined effect size is only some kind of mixed effect and does not necessarily have a causal explanation. It is difficult to produce identical results for the same research question.

Second, in the literature collection link, we made certain selections of the existing literature to ensure the reliability of the results. However, this prevents the inclusion of a large amount of literature and the study of all relevant literature individually, which will inevitably lead to relevant errors in the conclusion.

Third, the evaluation of students’ entrepreneurial abilities was based on their subjective perceptions, and there was a discrepancy between perceptions and facts. Obviously, the risk that students’ perceptions of the external world may differ from reality is common (Turker and Senem Sonmez, 2009), which reinforces the uncertainty of the data we collected.

In addition, it is not sufficient to adopt only the three dimensions of entrepreneurial activity in higher education. It is also necessary to pay attention to the impact of each component of each dimension on students’ entrepreneurial abilities because some elements in the sub-dimensions may have a negative impact on students’ entrepreneurship. If the positive and negative relationships between the factors are offset, the impact of college entrepreneurial activities on students’ entrepreneurial abilities may not be properly measured.

## Author contributions

JH, KZ, and SF designed the study, performed the research, analysed data, and wrote the manuscript.
